# Carotid Cavernous Fistula: A Case Report of a Rare Diagnosis and Presentation

**DOI:** 10.7759/cureus.117152

**Published:** 2026-09-29

**Authors:** Christian Cochrane, Hanna S Nour, Shayne Gue

**Affiliations:** 1 Emergency Medicine, HCA Healthcare, Kissimmee, USA; 2 Academic Affairs, University of Central Florida, Orlando, USA; 3 Emergency Medicine, BayCare Health System/St. Joseph's Hospital, Tampa, USA; 4 Medical Education, University of Central Florida College of Medicine, Orlando, USA

**Keywords:** basilar skull fractures, blurry vision, carotid-cavernous fistula, cavernous fistulas, cerebral aneurysms, connective tissue disorders, diplopia, ehlers-danlos, facial trauma, fibromuscular dysplasia

## Abstract

Periorbital swelling can differ from simple allergic conjunctivitis, orbital cellulitis, and cavernous sinus thrombosis, as well as rapidly expanding retrobulbar hemorrhage. An uncommon cause is the carotid-cavernous fistula (CCF), an abnormal arteriovenous connection between the carotid artery and the cavernous sinus. CCFs can cause periorbital swelling, diplopia, cranial nerve palsies, and ultimately vision loss secondary to retinal detachment, vitreous detachment, or intracranial hemorrhage. CCFs are strongly associated with previous head trauma, especially basilar skull fractures, and with connective tissue disorders. However, according to the patient, she had no history of connective tissue disorder or previous trauma. She presented to the clinic with right periorbital swelling, chemosis, and mild, painful extraocular movements of two months' duration and was referred to the emergency department for imaging. CT angiography (CTA) showed a right high-flow CCF. The patient was admitted for cerebral angiography and embolization. The procedure was successful, and her symptoms resolved completely.

## Introduction

A carotid-cavernous fistula (CCF) is defined as an abnormal arteriovenous communication between the carotid artery and the cavernous sinus. CCFs are classified by etiology (traumatic or spontaneous), hemodynamics (high- or low-flow), or anatomy (direct or indirect) [1]. Traumatic etiologies, including basilar skull fractures and iatrogenic injuries, are the most common causes of CCFs and typically present as direct, high-flow fistulas in young males. Although the overall incidence of CCFs is not well established, approximately 4% of basilar skull fractures result in CCFs [2]. Spontaneous CCFs are less common and generally occur in older females with predisposing genetic conditions such as Ehlers-Danlos syndrome (EDS) type IV and fibromuscular dysplasia [3].

The clinical presentation of CCFs varies depending on whether the fistula is direct or indirect. Direct CCFs involve arterial flow from the internal carotid artery (ICA) into the cavernous sinus and typically present acutely with symptoms such as proptosis, chemosis, orbital bruits, cranial nerve deficits, headache, diplopia, blurred vision, and orbital pain. In contrast, indirect CCFs are characterized by flow from branches of the ICA and external carotid artery (ECA) into the cavernous sinus, usually presenting with an insidious onset and most commonly with conjunctival injection.

Catheter cerebral angiography remains the gold standard for diagnosing CCFs, typically following initial evaluation with CT and MRI [4]. The primary treatment objective is to occlude the fistula while preserving normal ICA flow, with endovascular occlusion as the first-line therapeutic approach [5]. Notably, a spontaneous, high-flow CCF in the absence of trauma or a known connective tissue disorder, as seen in this case, is highly unusual and can initially lead to diagnostic confusion. This case report highlights the importance of considering this rare diagnosis even in the absence of classic risk factors in order to avoid delays in appropriate management.

## Case presentation

A 54-year-old female with no significant past medical history presented to the emergency department with right eye swelling that had been present for the past two months, along with worsening blurry vision and painful extraocular movements. The patient had initially visited her primary care physician and had been diagnosed with allergies; she had been prescribed a five-day course of prednisone and cetirizine, which she had completed without any improvement in her symptoms. She had then been referred to ophthalmology and prescribed steroid drops with a follow-up scheduled within one week; at the follow-up, her ophthalmologist had instructed her to go to the emergency department for imaging.

The patient denied fever, headache, unexplained weight loss, lightheadedness, nausea, vomiting, chest pain, or shortness of breath. The patient denied alcohol use, tobacco use, and illicit drug use. Her family history was significant for her father's death from a brain aneurysm. She denied any history of head trauma before the initial presentation. On physical examination, the patient's vital signs were within normal limits. Her ocular exam showed right periorbital swelling, mild proptosis, chemosis, a bruit, and mild pain with extraocular movements (Figure 1).

**Figure 1:**
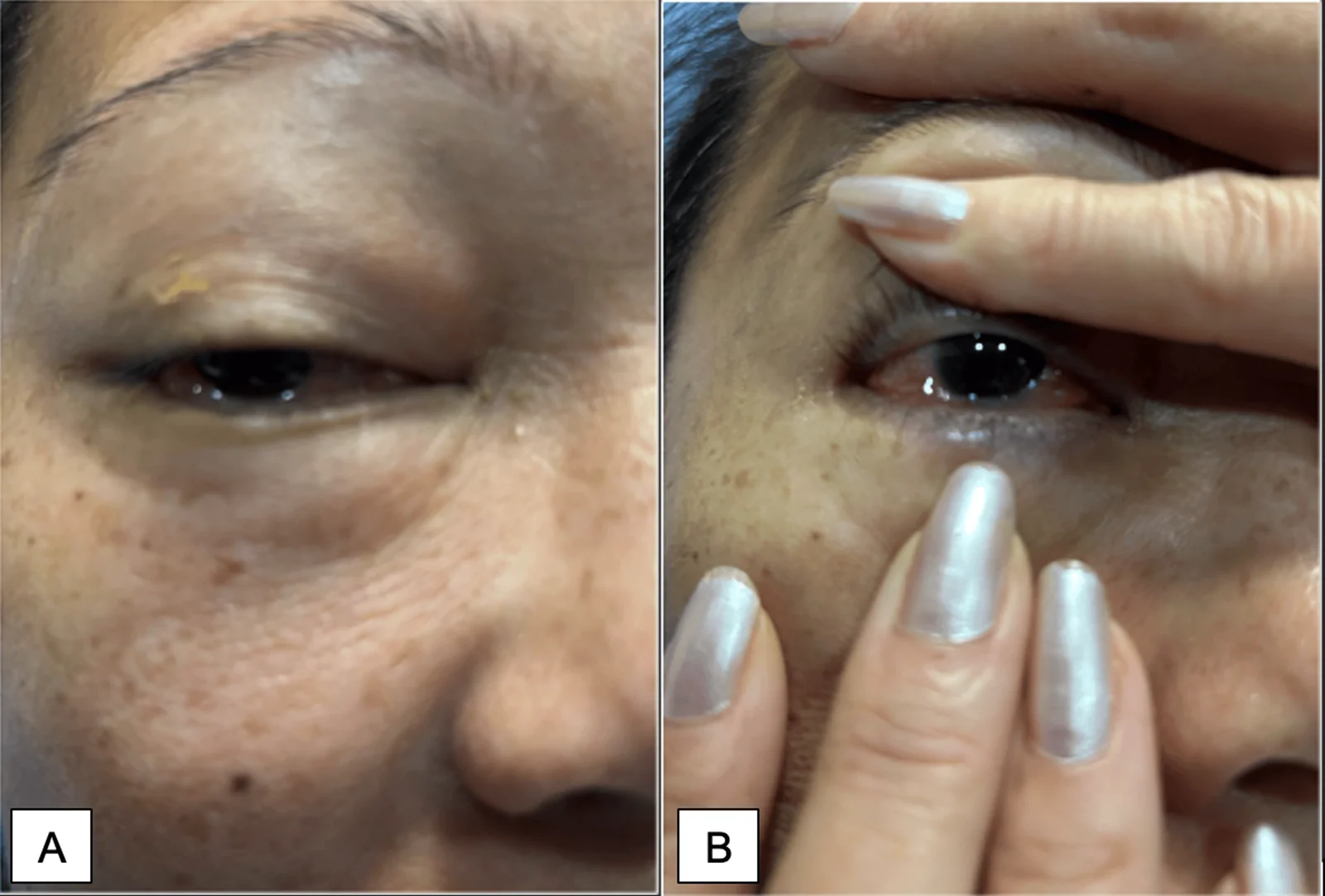
Patient images The photos show right eye periorbital swelling (A), chemosis (B), and decreased extraocular movements with pain

No cranial nerve deficits were noted. Visual acuity was 20/25 OD and 20/20 OS. The remainder of her physical exam was unremarkable, including normal strength and sensation. Reflexes were 2/4. Complete blood count (CBC) and basic metabolic panel (BMP) were within normal limits. Noncontrast CT of the head and brain was unremarkable; however, noncontrast maxillofacial CT showed right ophthalmic vein enlargement, suggesting a carotid-cavernous fistula (Figure 2). This finding prompted further evaluation with CT angiography (CTA) of the head and neck, with and without contrast, which confirmed early filling of the right cavernous sinus with arterial enhancement, dilation of the right ophthalmic vein, and multiple varices along the right side of the face, consistent with a CCF (Figure 3). Based on these findings, the patient was transferred to a tertiary comprehensive stroke center for neurologic evaluation and possible intervention. The patient was admitted to the medicine service, and both neurology and interventional radiology were consulted for further management.

**Figure 2:**
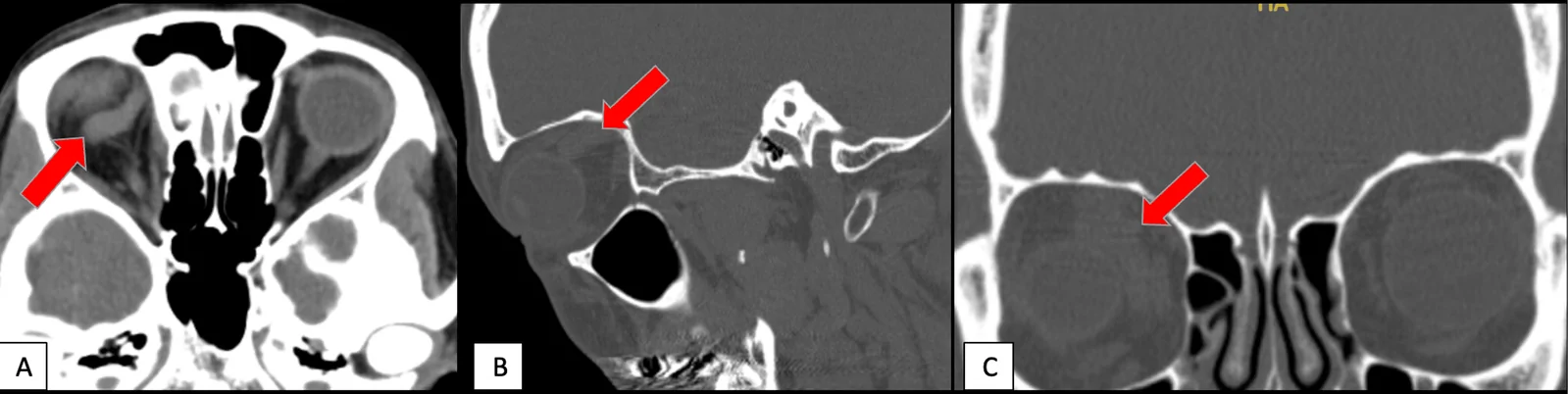
CT maxillofacial without contrast showing right eye ophthalmic vein enlargement (red arrow) Axial view (A), sagittal view (B), and coronal view (C) CT: computed tomography

**Figure 3:**
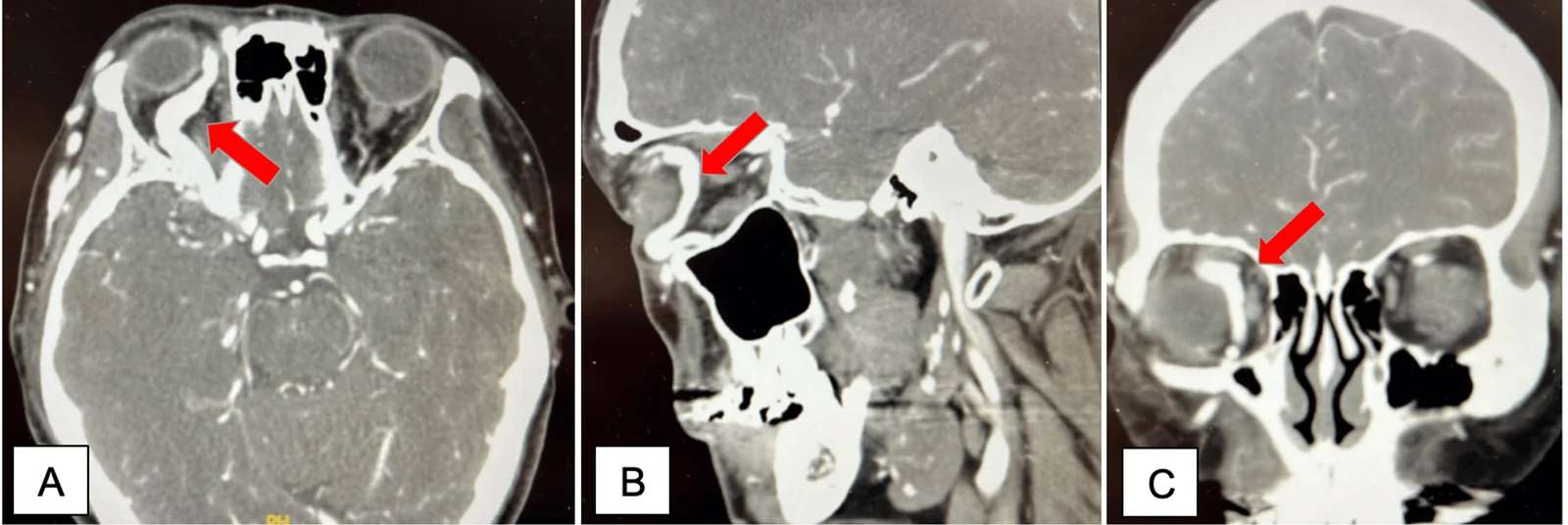
CTA brain with and without contrast showing a right high-flow carotid cavernous fistula (red arrow) Axial view (A), sagittal view (B), and coronal view (C) CTA: computed tomography angiography

The interventional neurology team performed cerebral angiography the following day, which revealed a high-flow, direct CCF between the right ICA and the right cavernous sinus, with rapid arterial flow into the cavernous sinus (Figure 4). They noted retrograde filling and marked dilatation of the right superior ophthalmic vein and right facial venous plexus. No branches other than the right ICA supplied the fistula. The right cavernous sinus was unable to be accessed for embolization due to the direction of flow and the tortuosity of the venous structures. A temporary balloon occlusion test was performed, and the patient had adequate collateral circulation around the right ICA. Therefore, a portion of the right ICA could be sacrificed across the fistula without neurological compromise. The team performed trans-arterial endovascular coiling with fifteen coils, achieving a 90% reduction in flow (Figure 5). The patient was admitted overnight to the ICU for close neurovascular monitoring. The patient had complete symptom resolution and was downgraded from the ICU the following day. Repeat angiography two days later showed complete occlusion of the CCF. The patient had complete resolution of her visual changes and right eye swelling.

**Figure 4:**
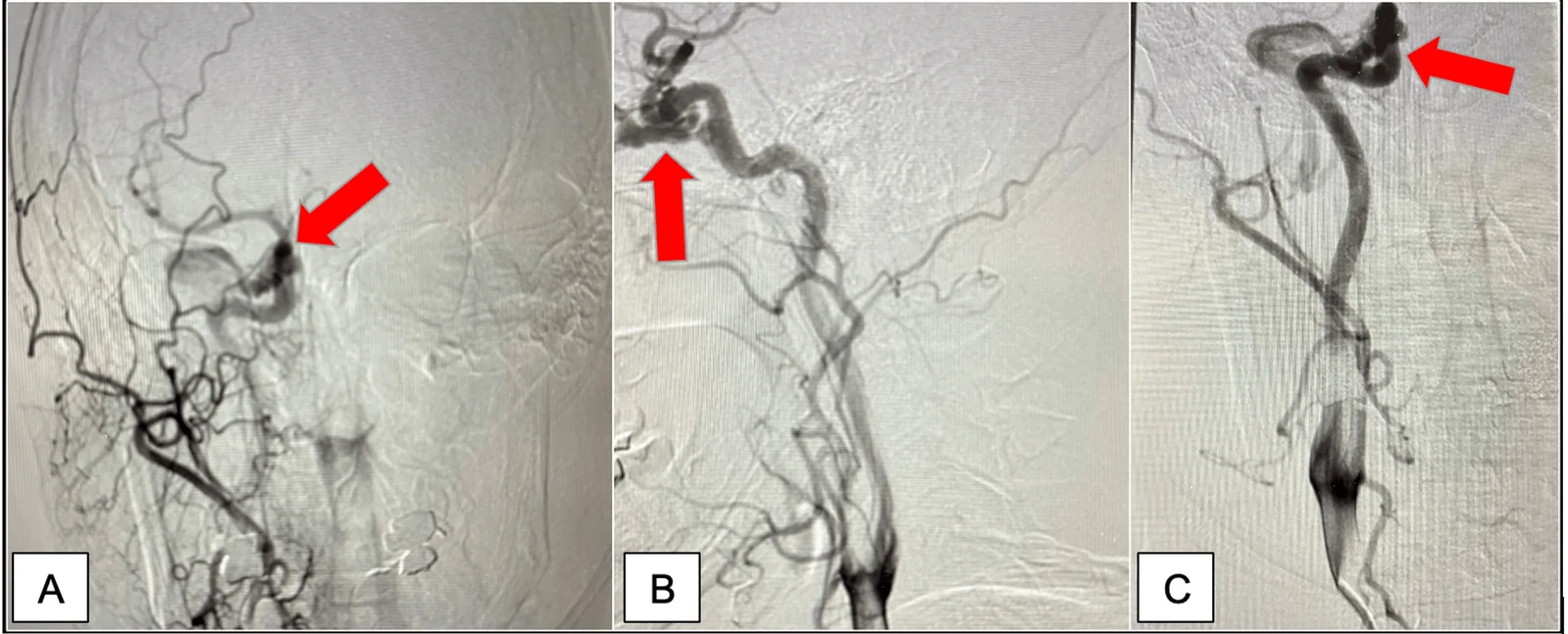
Initial cerebral angiography of right, direct, high-flow carotid-cavernous fistula (red arrow) Anterior coronal view (A), sagittal view (B), and magnified coronal view (C)

**Figure 5:**
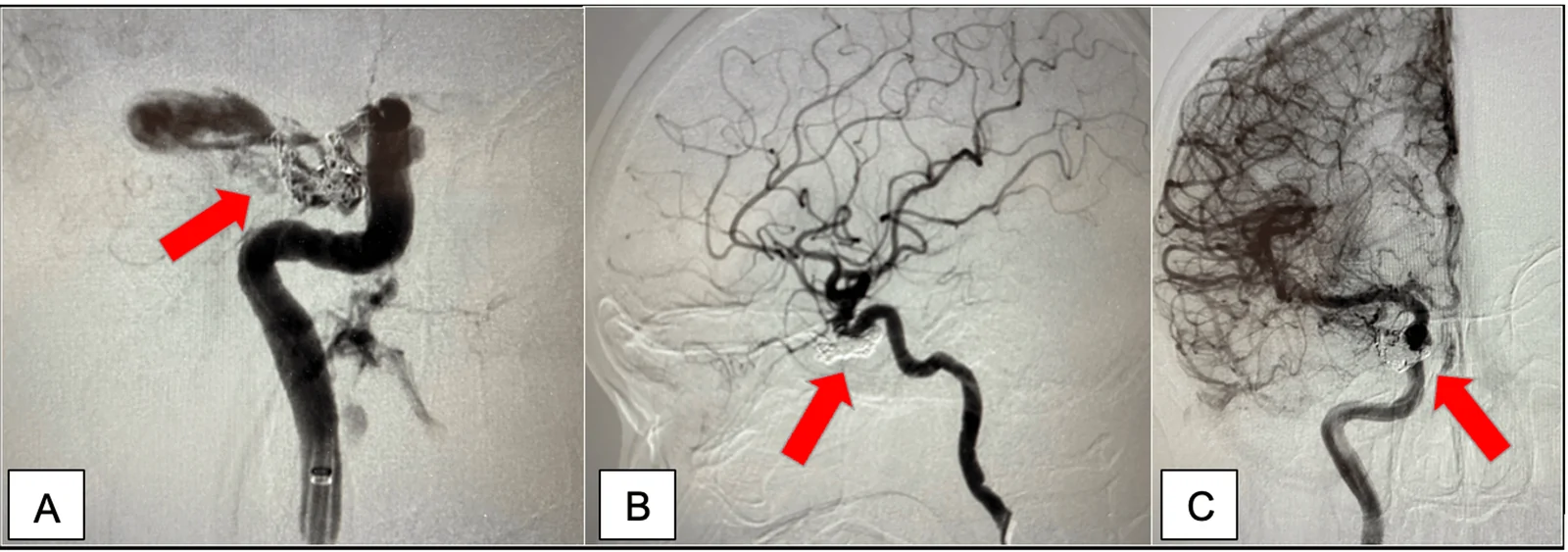
Post-coiling cerebral angiography of right, direct, high-flow carotid-cavernous fistula (red arrow) Magnified coronal view (A), sagittal view (B), and coronal view (C)

Further inpatient workup showed an elevated thyroid-stimulating hormone (TSH) and triiodothyronine (T3); however, thyroxine (T4) was normal. The patient also had elevated thyroid peroxidase (TPO) and thyroglobulin antibody levels. Endocrinology was consulted and determined that no treatment was needed at this time and recommended repeating the TSH in six weeks as an outpatient. She was also encouraged to follow up with dermatology on an outpatient basis to evaluate for an underlying connective tissue disorder that could have contributed to fistula formation.

## Discussion

Our patient was a 54-year-old woman with no significant past medical history who presented with a possible spontaneous CCF. Most patients who present with CCF have a recent history of facial or head trauma, which predisposes them to the condition secondary to small tears in the ICA from a possible shearing force mechanism; however, our patient denied any history of head trauma or skull fracture before her initial presentation. Our patient's father died of a cerebral aneurysm at 78 years old, which could be a result of a connective tissue disorder such as EDS type IV. However, neither she nor her father had been diagnosed with a connective tissue disorder, which is a common risk factor for CCFs. On physical exam, the patient had an orbital bruit (80% of patients), chemosis (55-89%), and proptosis (72-87%). However, she did not present with cranial nerve palsy, which can also be present in these patients: cranial nerve III (67%), IV (49%), and VI (50-85%) [6].

Although CCF is a rare condition, it should be included in the differential diagnosis for any emergency medicine physician providing care for a patient with unilateral periorbital swelling, regardless of whether there is a history of head trauma. Suspicion should be raised further if the patient has a recent history of basilar skull fracture, as this condition occurs in up to 4% of these patients [7]. Failure to diagnose a CCF could lead to acute angle-closure glaucoma, vitreous hemorrhage, retinal detachment, intracerebral hemorrhage, or subarachnoid hemorrhage, ultimately leading to vision loss or death.

## Conclusions

This case report highlights an uncommon yet serious diagnosis in a patient presenting with unilateral periorbital swelling. Our patient was a 54-year-old woman with no significant past medical history who presented to the emergency department with a two-month history of right periorbital swelling. She was diagnosed with a CCF and underwent cerebral embolization, resulting in complete resolution of her symptoms. This report further highlights the need for a comprehensive differential diagnosis and a broad diagnostic evaluation in patients presenting with these symptoms.
